# Can incorrect artificial intelligence (AI) results impact radiologists, and if so, what can we do about it? A multi-reader pilot study of lung cancer detection with chest radiography

**DOI:** 10.1007/s00330-023-09747-1

**Published:** 2023-06-02

**Authors:** Michael H. Bernstein, Michael K. Atalay, Elizabeth H. Dibble, Aaron W. P. Maxwell, Adib R. Karam, Saurabh Agarwal, Robert C. Ward, Terrance T. Healey, Grayson L. Baird

**Affiliations:** 1 Department of Diagnostic Imaging, Warren Alpert Medical School of Brown University, Providence, RI USA; 2 Rhode Island Hospital, Providence, RI USA; 3 Brown Radiology Human Factors Laboratory, Providence, RI USA

**Keywords:** Artificial intelligence, Psychology, Cognitive science

## Abstract

**Objective:**

To examine whether incorrect AI results impact radiologist performance, and if so, whether human factors can be optimized to reduce error.

**Methods:**

Multi-reader design, 6 radiologists interpreted 90 identical chest radiographs (follow-up CT needed: yes/no) on four occasions (09/20–01/22). No AI result was provided for session 1. Sham AI results were provided for sessions 2–4, and AI for 12 cases were manipulated to be incorrect (8 false positives (FP), 4 false negatives (FN)) (0.87 ROC-AUC). In the Delete AI (No Box) condition, radiologists were told AI results would not be saved for the evaluation. In Keep AI (No Box) and Keep AI (Box), radiologists were told results would be saved. In Keep AI (Box), the ostensible AI program visually outlined the region of suspicion. AI results were constant between conditions.

**Results:**

Relative to the No AI condition (FN = 2.7%, FP = 51.4%), FN and FPs were higher in the Keep AI (No Box) (FN = 33.0%, FP = 86.0%), Delete AI (No Box) (FN = 26.7%, FP = 80.5%), and Keep AI (Box) (FN = to 20.7%, FP = 80.5%) conditions (all *p*s < 0.05). FNs were higher in the Keep AI (No Box) condition (33.0%) than in the Keep AI (Box) condition (20.7%) (*p* = 0.04). FPs were higher in the Keep AI (No Box) (86.0%) condition than in the Delete AI (No Box) condition (80.5%) (*p* = 0.03).

**Conclusion:**

Incorrect AI causes radiologists to make incorrect follow-up decisions when they were correct without AI. This effect is mitigated when radiologists believe AI will be deleted from the patient’s file or a box is provided around the region of interest.

**Clinical relevance statement:**

When AI is wrong, radiologists make more errors than they would have without AI. Based on human factors psychology, our manuscript provides evidence for two AI implementation strategies that reduce the deleterious effects of incorrect AI.

**Key Points:**

• *When AI provided incorrect results, false negative and false positive rates among the radiologists increased.*

• *False positives decreased when AI results were deleted, versus kept, in the patient’s record.*

• *False negatives and false positives decreased when AI visually outlined the region of suspicion.*

**Supplementary Information:**

The online version contains supplementary material available at 10.1007/s00330-023-09747-1.

## Introduction

### The use of artificial intelligence (AI) in radiology

Artificial intelligence (AI) in radiology has increased dramatically over the past decade. While the first AI system was approved in 2008, only 6 received approval through 2014, and 141 were approved from 1/1/2020 to 10/11/2022 [1]. A 2020 survey of ACR members found that approximately 33% of radiologists now report using AI, with another 20% anticipating that they will adopt AI in the next 5 years [2].

### AI accuracy: What to do when AI is wrong?

In a review of 503 studies of AI algorithms, receiver operating characteristic area under the curve (ROC-AUC) for diagnostic imaging ranged from 0.864 to 0.937 for lung nodules or lung cancer on chest CT and chest X-ray examinations [3]. The sensitivity and specificity were 0.87 and 0.89, respectively, for chest x-ray abnormalities on these AI systems. A specificity of 0.89 means that **AI will return a false positive diagnosis for 11 out of 100 true negative cases**. More concerning, a sensitivity of 0.87 means that **AI will return a false negative diagnosis for 13 out of 100 true positive cases**. While radiologist performance improves when they are using versus not using AI [4–6], no AI system can be 100% accurate. We should always anticipate some false negatives and false positives.

What are the consequences of incorrect AI? Can it persuade a radiologist to make a wrong call when they would have otherwise made the right call? One study with CAD suggests this may be the case. Alberdi et al. [7] had two different sets of readers interpret mammograms; one set did so with the aid of CAD while another did not have CAD. When CAD feedback was a false negative, only 21% of readers in the CAD condition indicated cancer was present; 46% of these cases were accurately interpreted as a positive in the no CAD condition.

Another question remains: How can the deleterious effect of inaccurate feedback be prevented or mitigated by simply modifying how the AI feedback is presented? Answering this is consistent with the stated goals of the ACR’s Data Science Institute (DSI) to “facilitate the development and *implementation* (emphasis added) of AI applications that will help radiology professionals provide improved medical care” [8]. Indeed, while much focus has been placed on developing AI for radiologists*, research examining how AI could adversely impact radiologist decision making is severely lacking* (notwithstanding the aforementioned CAD study)*.* To improve patient outcomes, the AI algorithm, as well as the implementation of the AI, should both be optimized.

### Optimizing human factors in radiology

One field of study that can address these questions is human factors psychology—the study of interactions between humans and systems—here, AI [9]. A few studies have examined human factors in the context of radiology [10–13]. However, there are no published empirical studies examining human factors among radiologists *in the context of AI implementation*, although it has been broached in several theoretical papers [14–17].

### Current study

The present study, to our knowledge, is the first attempt to examine two related questions: (1) Do incorrect AI results deleteriously impact radiologist performance, and if so, (2) can human factors be optimized to reduce this impact? We focus on two key human factors with real-world implications: (1) whether AI results are kept or deleted in the patient’s file, and (2) whether AI does or does not visually outline the area of suspicion. Hypotheses are presented in Table 1.

**Table 1:** Study hypotheses

#	Hypothesis
1	AI feedback that a positive case is negative will cause an increase in false negatives
2	The effect described in hypothesis 1 will be exacerbated when radiologists believe AI is included in the patient record versus not
3	AI feedback that a negative case is positive will cause an increase in false positives
4	The effect described in hypothesis 3 will be exacerbated when radiologists believe AI is included in the patient record versus not
5	Providing a region of interest (box) where AI reports that the cancer is will decrease the effect described in hypotheses 1 and 3
6	AI will increase radiologist’s confidence
7	Providing a region of interest will exacerbate the effect described in hypothesis 6

## Materials and methods

### Design

**Fig. 1:**
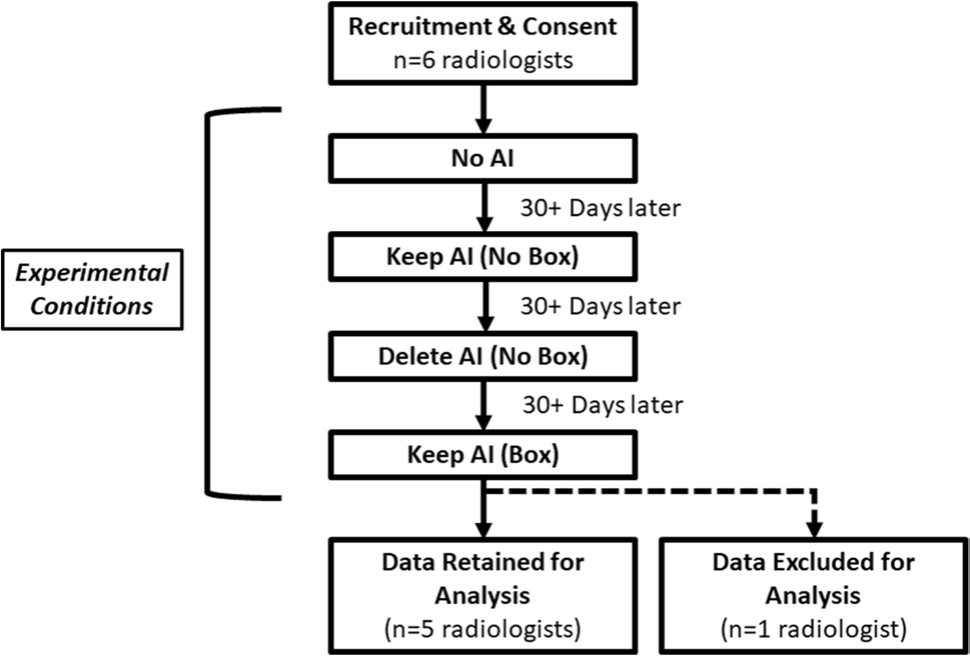
Study flow chart. Overall experimental procedure is displayed. The order of Keep AI (No Box) and Delete AI (No Box) was counterbalanced such that *n* = 3 participated in Keep AI (No Box) first, and *n* = 2 participated in Delete AI (No Box) first

A fully crossed, complete, and balanced block design was used where all radiologists read the same 90 chest x-rays from 90 patients across four different conditions (No AI, AI results Keep (No Box), AI results Delete (No Box), AI results Keep (Box)) (Fig. 1). The order of conditions AI Keep (No Box) and AI Delete (No Box) were randomly counterbalanced between radiologists to reduce an order effect. No AI was always first and Keep AI (Box) was always last to ensure no contamination (i.e., the first condition was standard of care and provided no feedback to contaminate future conditions, the last condition pinpointed suspected pathology). The AI results were manipulated to provide 8 false positives (FPs) and 4 false negatives (FNs). It was correct for the remaining 78 cases (ROC-AUC of 0.87). Cases were ordered randomly. Radiologists were blinded to individual patient identifying information and reading sessions were separated by at least 1 month (average inter-session duration of included radiologists is 113.8 days).

### Cover story

Radiologists were told that the goal of the study was to look at several different AI programs that have been developed for chest radiography. As such, radiologists were told they would interpret radiographs on several different trials. Radiologists were told they should indicate whether follow-up imaging was needed for a possible pulmonary lesion. Unbeknownst to the radiologists, the only pathology present was lung cancer. For one trial, radiologists were told they would not have AI (No AI) so they could serve as the control group for that AI system. For each of the other sessions, radiologists were told they would be interpreting cases with a unique AI program.

### AI feedback

The AI results for each image were provided in two ways. First, either “Abnormal” or “Normal” was shown visually in the top right-hand corner of the image. Second, the experimenter read the AI results aloud to reinforce the Keep vs. Delete manipulation (Supplement 1). For the **AI Keep (Box)** condition, a box was placed when the AI result was “abnormal.” No such box was used for the other conditions. Because the effect of AI feedback implementation was of interest, AI feedback needed to be controlled. Thus, no real AI systems were actually used. The AI feedback for each image was chosen by MKA. This enabled us to control the number of true positives (TPs), true negatives (TNs), FPs, and FNs, and which cases fell into each of these categories. The AI result was identical between experimental conditions; thus, only the instructions regarding keep vs. delete vs. box differed, (Supplement 1).

Radiologists were told the ROC-AUC value of each system was higher than 0.85, which approximates clinical practice [3] and was true for the simulated AI systems.

### Case selection

A total of 90 frontal chest radiographs (CXR) (50% positive for lung cancer) taken from patients imaged between 2013 and 2016 were used. Positive CXRs were obtained within 2 weeks of a CT demonstrating a single dominant lung nodule/mass confirmed at biopsy to be lung cancer and NO other airspace opacities, consolidations, or macronodules detectable at CXR; negative CXRs were obtained within 2 weeks of a CT demonstrating NO nodule/mass. More information is provided in supplement 2.

### Procedure

**Fig. 2:**
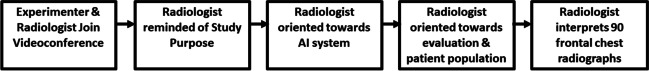
Procedural overview. A brief description of the major procedural elements within each session is shown

Due to COVID-19, the experimenter and radiologist communicated over videoconferencing. A convenience sample comprising six radiologists consented and participated (E.H.D., A.W.P.M., A.R.K., S.A., R.C.W., T.T.H.) with 3, 1, 14, 6, 5, and 12 years of experience, respectively. At the appointed time, the radiologist and experimenter (M.H.B.) joined a videoconference. Radiologists were reminded about the ostensible goal of the experiment (evaluating several different AI programs). Radiologists were then told that the ROC-AUC for the company’s AI program in the present session was higher than 0.85 (sessions 2–4). To encourage effortful participation, radiologists were told that their performance relative to the other radiologists would be evaluated by M.H.B. and M.K.A. (Vice Chair of Research and thoracic radiologist) upon conclusion of the study (though there was no such evaluation in reality). Radiologists viewed a sample evaluation sheet. Radiologists were told that in order to fully evaluate each AI system, all images were taken from 55–75-year-old smokers who were outpatients, so the dataset was therefore enriched. In actuality, as noted above, the ratio of positive and negative cases was equal though radiologists were not told prevalence rate to avoid confounding their performance [18]. No history was provided for any of the images. Next, the radiologist interpreted each anonymized image one-by-one using an ORTHANC-based research PACS [19]. All image interpretation was conducted in a clinical radiology reading room using standard PACS monitors. Except for the manner in which cases were loaded from Orthanc, viewing conditions were identical to those found in clinical practice. The experimenter was blinded to ground truth. The procedure is shown in Fig. 2.

### Interpretation and outcomes

Radiologists responded to two questions for each image: (1) whether they recommend follow-up CT imaging (yes/no) and (2) their level of confidence (1 = not at all to 5 = very confident). All responses were read aloud and transcribed by the experimenter. Follow-up imaging, as a clinical decision, was used as the primary outcome. Thus, a failure to follow up a positive is a FN, not following up a negative is a TN, following up a positive is a TP, and following up a negative is a FP.

### Debriefing and exclusion

Following the conclusion of the study, radiologists were debriefed, and all deception was explained. One radiologist was excluded from analyses, so analyses were performed on the remaining 5 radiologists. The excluded radiologist did not fully believe the cover story, and correctly thought the experimenter might have been interested in his/her behavior. He/she also reported being unaware of the keep versus delete manipulation. All procedures were approved by the Rhode Island Hospital IRB.

### Statistical analysis

All analyses were conducted using SAS Software 9.4 (SAS Inc.). FPs and FNs were examined between conditions to test the hypotheses using generalized linear mixed modeling (GLMM) assuming a binary distribution. GLMM was used to examine diagnostic confidence between conditions. Alpha was established a priori at the 0.05 level and all interval estimates are calculated for 95% confidence. Analyses were conducted by the statistical author (G.L.B.).

### Manipulations

The AI result manipulations (Keep vs. Delete; Box vs. No Box) were introduced at three points during the experiment: (1) at the beginning of the session during preliminary instructions, (2) when the experimenter provided oral AI results, and (3) during the sample evaluation sheet. These are detailed in Supplement 1. Briefly, in **AI Delete (No Box)**, radiologists were told AI results would not be saved. In **AI Keep (No Box)** and **AI Keep (Box)**, radiologists were told the results of AI would be saved. **AI Keep (Box)**, unlike both other AI conditions, provided a box around suspicious regions when AI indicated it was abnormal.

## Results

### Patient characteristics

Patients were a median of 68 years old (IQR = 62–77). In total, 53.3% identified as female and 46.7% identified as male. Regarding race, 81.1% were White/Caucasian, 7.8% were Black/African American, 1.1% were Asian, 1.1% were Native Hawaiian/other Pacific Islander, and 8.9% reported their race as “other.” Regarding ethnicity, 8.9% were Hispanic and 91.1% were non-Hispanic.

### False negatives

**Fig. 3:**
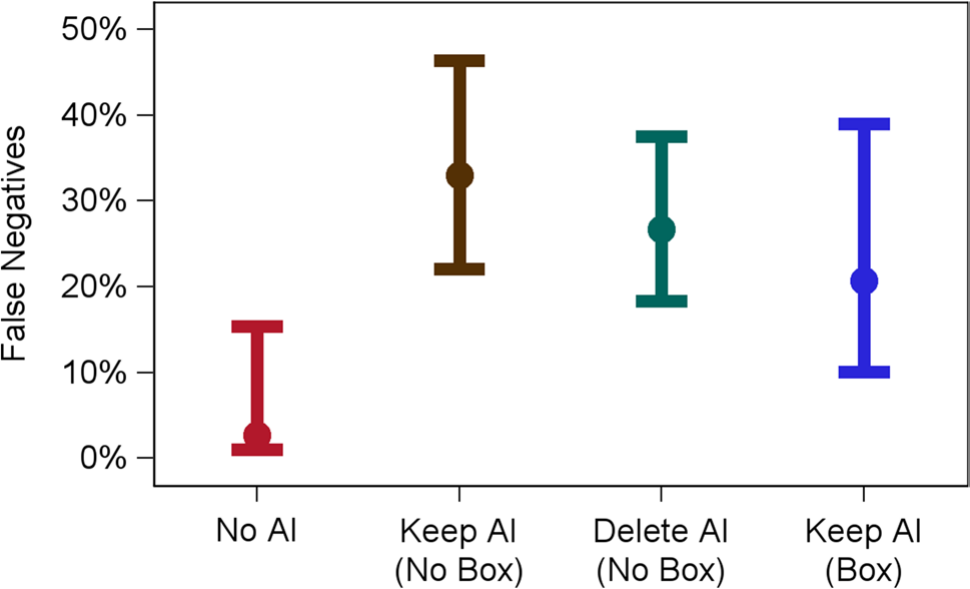
False negatives (incorrect AI feedback) by experimental condition. False negative percent (*y*-axis) is shown for the No AI (red), Keep AI (No Box) (brown), Delete AI (No Box) (green), and Keep AI (Box) (blue) conditions (*x*-axis). Mean (circle) and 95% confidence intervals are displayed. Results display the four conditions for the 4 cases where AI provided false negative feedback

As anticipated in hypothesis 1, incorrect AI results that a true pathology positive case was “normal” increased false negatives (Fig. 3). We focus below on the 4 cases where AI was manipulated to have false negative AI results. Among these cases in the No AI condition, the false negative percent was 2.7% (95% CI (0.4, 16.0)); false negatives increased to 33.0% (95% CI (21.5, 46.9)) in the AI Keep (No Box) condition (*p* = 0.006), increased to 26.7% (95%CI (17.7, 38.1)) in the AI Delete (No Box) condition (*p* = 0.009), and increased to 20.7% (95% CI (9.4, 39.6)) in the AI Keep (Box) condition (*p* = 0.02).

As anticipated in hypothesis 2, this effect was higher in the AI Keep (No Box) versus AI Delete (No Box) condition (33.0% vs. 26.7%), though this difference failed to achieve significance (*p* = 0.13). As anticipated in hypothesis 5, FNs were higher in AI Keep (No Box) than in AI Keep (Box) (33.0% vs. 20.7%, *p* = 0.04).

### False positives

**Fig. 4:**
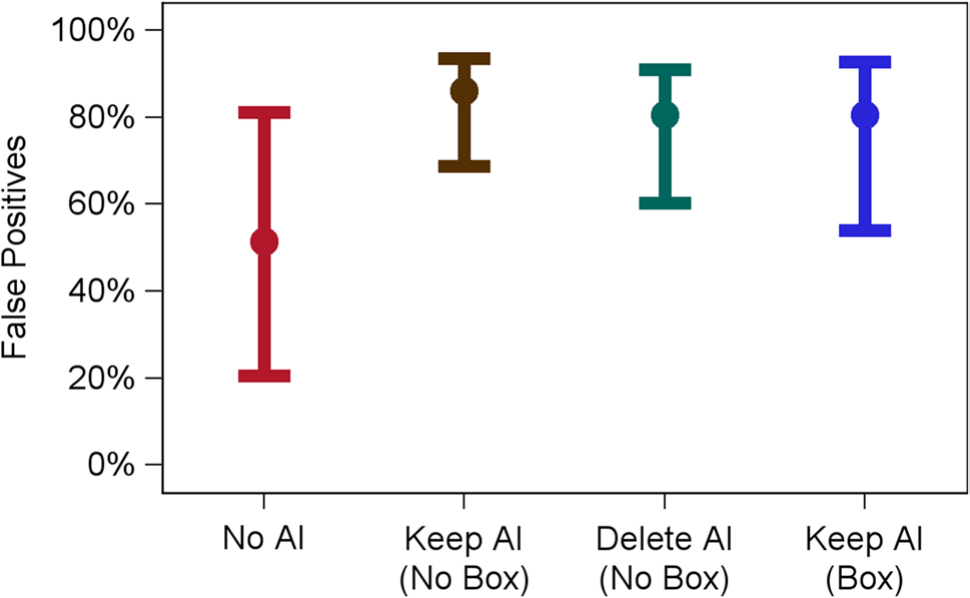
False positives (incorrect AI feedback) by experimental condition. False positive percent (*y*-axis) is shown for the No AI (red), Keep AI (No Box) (brown), Delete AI (No Box) (green), and Keep AI (Box) (blue) conditions (*x*-axis). Mean (circle) and 95% confidence intervals are displayed. Results display the four conditions for the 8 cases where AI provided false positive feedback

As anticipated in hypothesis 3, incorrect AI results that a true pathology negative case was “abnormal” increased false positives (Fig. 4). We focus below on the 8 cases where AI was manipulated to have false positive results. Among these cases in the No AI condition, the false positive percent was 51.4% (95% CI (19.4, 82.3)); false positives increased to 86.0% (95% CI (67.5, 94.8)) in the AI Keep (No Box) condition (*p* = 0.01), increased to 80.5% (95% CI (59.0, 92.2)) in the AI Delete (No Box) condition (*p* = 0.008), and increased to 80.5% (95% CI (52.7, 93.8)) in the AI Keep (Box) condition, though this did not quite achieve significance (*p* = 0.052).

As anticipated in hypothesis 4, false positives were higher in the AI Keep (No Box) condition versus the AI Keep (Delete) condition (86.0% vs. 80.5%, *p* = 0.03). As anticipated in hypothesis 5, false positives were higher in the AI Keep (No Box) condition versus the AI Keep (Box) condition (86.0% vs. 80.5%), though this did not achieve significance (*p* = 0.19), and it had an identical value to the Delete (No Box) condition (i.e., both 80.5%).

### Confidence

There were minimal effects regarding confidence, not as anticipated in hypotheses 6–7. These are explained in detail in Supplement 3.

### True positives and negatives

As anticipated, when AI provided correct positive and correct negative results, radiologists were more accurate in all AI conditions compared to the no AI condition. Furthermore, TPs were higher in the AI Keep (Box) condition compared to the AI Keep (No Box) condition (*p* < 0.001). Full results are described in Supplement 4.

## Discussion

As the adoption of AI in radiology continues to proliferate, it is imperative to consider **how** AI should be incorporated into a clinical setting. This issue would not be important if AI were always correct; in the theoretical case where AI’s false negative and positive rates achieve 0%, the human factors of AI implementation would be irrelevant. However, AI implementation matters **precisely because** AI is stochastic and imperfect [3]. While radiologists should use AI feedback as supplemental information, akin to laboratory values, one important role of the radiologist, particularly in the coming years, is to determine how to integrate possibly erroneous AI results into their final report. To echo the ACR, “AI is never ‘in place of’ or ‘an alternative to’ radiologists — [radiologists] will have a key role in the supervision of AI to ensure patient safety” [20].

### Can incorrect AI lead the radiologist astray?

As predicted, in the present study, we observed that incorrect AI results can cause radiologists to make incorrect decisions when they would have otherwise been correct. For instance, when no AI was provided, false negatives were 2.7%. But when AI provided false feedback that there was no abnormality, false negatives increased to 20.7–33.0%, depending on the AI results condition. Similarly, when no AI was provided, false positives were 51.4%. But when AI provided incorrect results that there was an abnormality, false positives increased to 80.5–86.0%. Our results are similar to a prior study [7] showing that incorrect CAD results hurt radiologist’s performance, although only our study included a within-subjects design to control for the effect of the radiologist. Our study is consistent with the notion of “appeal to authority” from logic and cognitive science [21] where a person is influenced by authority. Presumably, the more accurate an AI system is perceived, the more a radiologist will be influenced by incorrect feedback from that system.

### Can the impact of incorrect AI be mitigated depending on the human factors of AI implementation?

There was evidence that the way AI was implemented impacted radiologist performance. Providing a box around the region of interest resulted in fewer false negatives (33.0% vs. 20.7%) and a non-significant trend towards fewer false positives (86.0% vs. 80.5%) than did the absence of a box. The fact that a box reduced false negatives from 33.0 to 20.7% may be driven by differences in cognitive load. When radiologists interpret positive cases with a box, they might have more cognitive resources to carefully search ostensibly negative cases and contradict AI when AI is wrong. Said differently, providing a box around suspicious regions may mitigate fatigue during a reading session by reducing the region needed to visually search an image, thereby enhancing performance even on cases where no box is provided [22]. Also, the false positives were higher when the AI results were “kept” versus “deleted.” Radiologists were less likely to disagree with AI (even when AI is incorrect) if there is a record of that disagreement occurring. We would assume that the safe option from a discovery and liability standpoint is to agree with AI when the AI is on record.

### Limitations

Most importantly, we could not realistically emulate the real-world consequences from AI results being kept or deleted in a patient’s file thereby limiting ecological validity. Unlike clinical practice, radiologists in this experiment knew they were participating in research without the possibility of any real legal (discoverability), financial, ethical, and psychological repercussions to making a mistake—a lack of ecological validity.

Cases were read in an artificial setting with only frontal views (unlike clinical practice where lateral views may also be available). Participants comprised a small number of radiologists at one site. There was no AI Delete (Box) condition. Radiologists only indicated whether or not follow-up was needed. Thus, when a case was positive, and a radiologist said it was positive, we technically cannot be certain they focused on the correct region. However, all positive cases contained only one lesion. Although lateral views are valuable [23] and may have been available in some of these cases, for this study, radiologists only received frontal CXRs so that the imaging was standardized across all 90 cases.

Condition order was not fully counterbalanced. We used an enriched dataset consisting of more pathology than typically found in clinical practice, although this was practically needed to have enough positive cases and is consistent with other reader studies [24, 25]. Radiologists often interpreted cases during days devoted to administrative work or research, and therefore were probably less fatigued than during a clinical shift. A sham AI system was used rather than a real AI system. Finally, to show that radiologists could be misled (and that this could be mitigated), we had to select cases that could be manipulated to be wrong. In so doing, we manipulated AI for cases where the radiologists were *more likely* to be correct, and thus were presumably *less* effected by incorrect AI. This limitation, along some others discussed above related to the artificiality of the setting, probably *blunted* the true effect of our manipulations and thus shows the robustness of our results. If true, this would mean that the effects observed in this study are *actually larger in clinical* practice.

### Conclusion and implications

AI is often right but sometimes is wrong. Since we do not know when it is accurate, we must consider how to minimize the extent to which radiologists are influenced by incorrect results. In this study, we show that incorrect AI results can influence a radiologist to make a wrong decision. However, this effect is mitigated when radiologists are told the AI results are deleted, versus kept, in the patient’s file, and when AI provides a box that visually outlines suspicious regions. In fact, AI that included a box improved radiologists’ performance *when AI was both correct and incorrect*.

This study offers compelling initial evidence that human factors of AI can impact radiologists. To enhance patient care, radiology practices should consider how AI is implemented. Radiological societies should formulate guidelines for radiologists regarding the integration of AI results into the reporting of examinations. Moreover, radiologists should be trained in best practices for using AI tools clinically.

### Supplementary Information

Below is the link to the electronic supplementary material.

Supplementary file1 (PDF 306 kb)

## Abbreviations

ACR: American College of Radiology
AI: Artificial intelligence
CAD: Computer-aided detection
CXR: Chest radiograph
DSI: Data Science Institute
FN: False negative
FP: False positive
GLMM: Generalized linear mixed modeling
PACS: Picture archiving and communication system
TN: True negative
TP: True positive

## Declarations

### Guarantor

The scientific guarantor of this publication is Michael Bernstein.

### Statistics and biometry

One of the authors has significant statistical expertise.

### Methodology

• prospective

• experimental

• performed at one institution
