# Supplementary material for: Can incorrect artificial intelligence (AI) results impact radiologists, and if so, what can we do about it? A multi-reader pilot study of lung cancer detection with chest radiography
Source: Eur Radiol. 2023 Jun 2;33(11):8263–9. doi: 10.1007/s00330-023-09747-1 (PMC10235827; doi:10.1007/s00330-023-09747-1)

ELECTRONIC SUPPLEMENTARY MATERIAL

Can incorrect Artificial Intelligence (AI) results impact radiologists, and if so, what can we do about it? A multi-reader pilot study of Lung Cancer Detection with Chest Radiography

Supplement 1

Session Instructions

During the beginning of the session, when the experimenter was orienting the radiologist to their task, condition was manipulated in the following manner: In the No AI condition, radiologists read each image without the aid AI, so that they could supposedly serve as the control for that AI system. Then, in all AI sessions, radiologists were told that the AI algorithm had calculated whether the image is “normal” or “abnormal”. Further, in the AI Delete (No Box) condition, radiologists were told “The company who made this AI system deletes the AI feedback. In clinical use, this means it would not be kept with the patient’s file. It is there to help you but will be deleted after each read.” In the AI Keep (No Box) and AI Keep (Box) conditions, radiologists were told “The company who made this AI system keeps the AI feedback. Therefore, we are going to keep your responses as well as the AI feedback [including the box around the suspicious region: phrase added for AI Keep (Box) only] associated with each image. In clinical use, this means it would be kept in the patient’s file.” In the AI Keep Box condition, radiologists were also told “When the system identifies an abnormality, it will draw a box around the most suspicious region.”

Oral AI Feedback

As noted in the procedure subsection, the experimenter read the AI feedback aloud to the radiologist. For several of the cases in the AI Keep (No Box) and AI Keep (Box) conditions, he said “AI in the patient’s file says ‘normal/abnormal.’” In the AI Delete (No Box) condition, he said “AI says ‘normal/abnormal’, though it won’t be saved in the patient’s file” for several of the cases.

AI Evaluation Sheet

Also as noted in the procedure subsection, radiologists viewed a sample evaluation sheet during each session. The evaluation sheet was largely used to make the evaluation more salient. It therefore included one column showing ground truth, and one column showing the radiologist interpretation, with incorrect radiologist responses flagged in red font. Each row corresponded to one image.

However, another purpose was to further reinforce the manipulations. Thus, there was also a column labeled “AI feedback.” In the AI Keep (Box) and AI Keep (No Box) conditions, this column was populated with AI interpretations, and incorrect AI responses were flagged in red font. In the AI Delete (No Box) condition, rather than showing AI interpretations, the phrase “not recorded” was provided for all rows.

AI Keep (Box) Condition

For TP cases, a rectangular box was placed around the tumor. For FP cases, the box was positioned in a region of lung that *could* be interpreted as abnormal due to vessel crowding or superimposition of normal shadows. All boxes were roughly the same size, nearly square, spanned approximately 1/6th to 1/4th of one lung, and had a thin white border.

Supplement 2

MKA examined and carefully curated 90 frontal chest radiographs (CXR) (50% positive for lung cancer) from our imaging database using the following selection criteria: To show that AI could influence a radiologist to be incorrect when they had otherwise been correct, cases where radiologists were more accurate as a group during the No AI condition were considered as possibilities for providing incorrect AI results. From these, MKA selected 4 positive cases in which the nodules were small and/or subtle (juxtahilar, for example) such that AI could reasonably say ‘normal’ and 8 negative cases in which vessel crowding or superimposition of normal shadows could reasonably be misinterpreted by AI as ‘abnormal’ without drawing suspicion.

Supplement 3

Confidence, False Negatives*.* As partly anticipated in hypothesis 6, incorrect AI feedback that a true pathology positive case was “normal” increased confidence, but only for one condition (Figure 3). Specifically, without any AI feedback, radiologists’ confidence was 4.3 (95% CI [3.1, 4.8] on a 1-5 scale); this increased to 4.6 (95% CI [3.9, 4.9]) in the AI Delete (No Box) condition ($p<0.0001$). When radiologists were told the AI feedback was kept in the patient’s file (AI Keep [No Box]), this increase failed to be different then the No AI condition (4.4, 95% CI [3.8, 4.7] vs. 4.3, p=0.70). Likewise, not as anticipated (hypothesis 7), when radiologists were provided a box around the suspected area and told the AI feedback was retained in the patient’s file (Keep AI [Box]), this increase failed to be different compared to the No AI condition (4.4, 95% CI [3.6, 4.8] vs. 4.3, p=0.22).

Confidence, False Positives. Not as anticipated in hypothesis 6, there was no evidence that incorrect AI feedback of a true pathology negative case as “abnormal” increased confidence. Specifically, without any AI feedback, radiologist confidence was 3.9 (95% CI[3.0, 4.5] on a 1-5 scale); this failed to increase when radiologists were told the AI feedback was deleted in the patient’s file (AI Delete [No Box]) (3.8, 95% CI[3.3, 4.3], p=0.36) and when radiologists were told the AI feedback was kept in the patient’s file (AI Keep [No Box]) (4.0, 95% CI[3.6, 4.3], p=0.72). Likewise, not as anticipated (hypothesis 7), when radiologists were provided a box around the suspected area and told the AI feedback was kept in the patient’s file (AI Keep [Box]), this increase failed to be different compared to the No AI condition (3.8, 95% CI [3.1, 4.3], p=0.40).

Supplement 4

True Negatives

As anticipated, correct AI results that a true pathology negative case was "normal" increased the percent of true negatives (Figure S3a). In the No AI condition, the percent of true negatives was 77.3% (95% CI [65.6, 85.9]); the percent of true negatives increased to 89.7% (95% CI[85.5, 92.7)]) in the AI Keep (No Box) condition (p=0.01), increased to 90.7% (95%CI [82.4, 95.3]) in the AI Delete (No Box) condition (p=0.003), and increased to 90.7% (95% CI[83.8, 94.8]) in the AI Keep (Box) condition, (p= 0.008).

However, there was no evidence that this effect varied between how AI results were presented: AI Keep (No Box) versus AI Delete (No Box) condition (89.7% vs. 90.7%, p= 0.99), AI Keep (No Box) versus AI Keep (Box) (89.7% vs. 90.7%, p= 0.97) and AI Delete versus AI Keep (Box) (90.7% vs. 90.7%, p= 0.99).

True Positives

As anticipated, correct AI results that a true pathology positive case was "abnormal" increased the true positive percent (Figure S3b). In the No AI condition, the percent of true positives was 88.3% (95% CI [81.6, 92.8]); the percent of true positives increased to 95.1% (95% CI[89.5, 97.8)]) in the AI Keep (No Box) condition (p<0.001), increased to 94.7% (95%CI [86.2, 98.1]) in the AI Delete (No Box) condition (p=0.04), and increased to 97.8% (95% CI[94.6, 99.1]) in the AI Keep (Box) condition, (p<0.001).

Although there was no evidence that this effect varied between AI Keep (No Box) versus AI Delete (No Box) condition (95.1% vs. 94.7%, p= 0.98), the percent of true positives increased for AI Keep (Box) versus AI Keep (no Box) (97.8% vs. 95.1%, p<0.001) and versus AI Delete (97.8% vs. 94.7%, p<0.001).

Confidence for Correct AI Results

True Negatives.

As anticipated, correct AI results that a true pathology negative case was "normal" increased diagnostic confidence relative to No AI results (Figure S3c). Specifically, in the no AI condition radiologists' confidence was 4.26 (95% CI [3.9, 4.5] on a 1-5 scale); this increased to 4.6 (95% CI [4.3, 4.8]) in the AI Delete (No Box) condition (p=0.004). When radiologists were told the AI results was kept in the patient's file (AI Keep [No Box]), this increases to 4.6 (95% CI [4.2, 4.8]) p=0.02. Likewise, when radiologists were provided a box around the suspected area and told the AI results was retained in the patient's file (Keep AI [Box]), this increases to 4.7 (95% CI [4.2, 4.9]), p=0.02. However, there was no evidence that confidence varied between how AI results were presented (all p>0.05).

True Positives.

In the no AI condition, radiologists' diagnostic confidence was 4.7 (95% CI [4.3, 4.9] on a 1-5 scale); this increased to 4.8 (95% CI [4.6, 4.9]) in the AI Delete (No Box) condition (p=0.02) (Figure S3d). When radiologists were told the AI results were kept in the patient's file (AI Keep [No Box]), confidence was 4.7 (95% CI [4.4, 4.8]), which failed to be different p=0.86 from no AI. Likewise, when radiologists were provided with a box around the suspected area and told the AI results were retained in the patient's file (Keep AI [Box]), confidence was 4.6 (95% CI [4.2, 4.8]), which failed to be different from no AI, p=0.66.There was no evidence that confidence varied between how AI results were presented (all p>0.05), except confidence was higher when radiologists thought AI results were deleted from the patients file (4.8) compared with retained (4.6), p=0.03).

Figure S1. Confidence: False negative (incorrect AI feedback).
All cases were positive, although AI provided false negative feedback. Confidence rating (from 1=not at all confident to 5=very confident) are shown for the No AI (Red), Keep AI (No Box) (Brown), Delete AI (No Box) (Green), and Keep AI (Box) (Blue) conditions (x-axis). Mean and 95% Confidence Intervals are displayed.

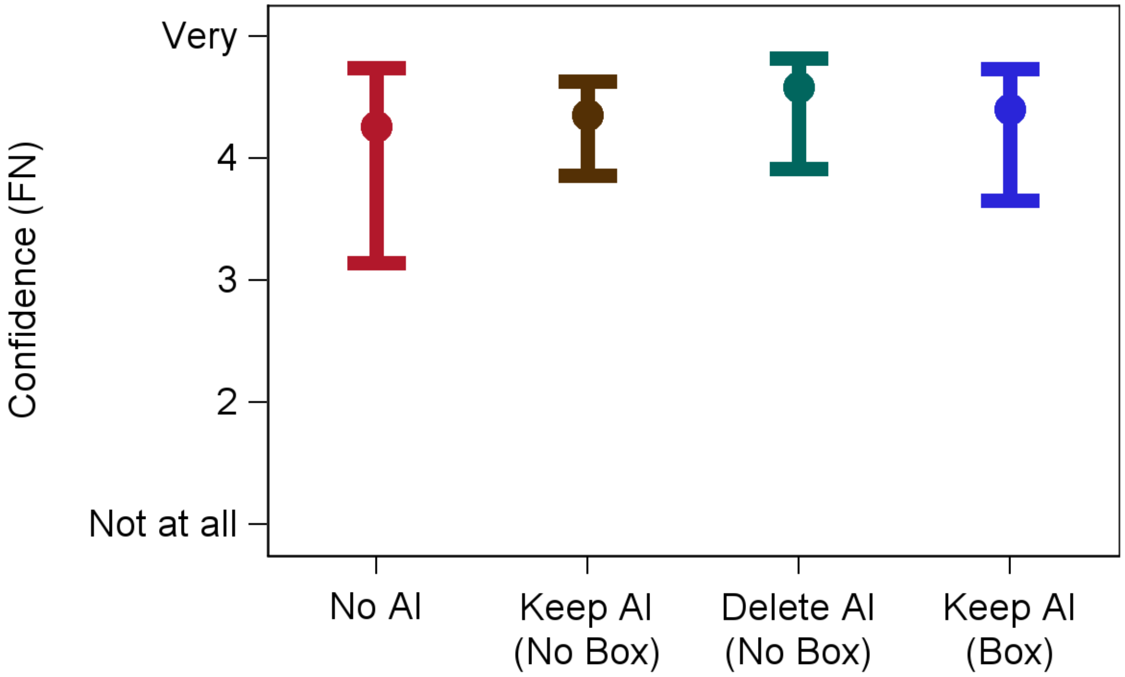

Figure S2. Confidence: False positive (incorrect AI feedback).
All cases were negative, although AI provided false positive feedback. Confidence rating (from 1=not at all confident to 5=very confident) are shown for the No AI (Red), Keep AI (No Box) (Brown), Delete AI (No Box) (Green), and Keep AI (Box) (Blue) conditions (x-axis). Mean and 95% Confidence Intervals are displayed.

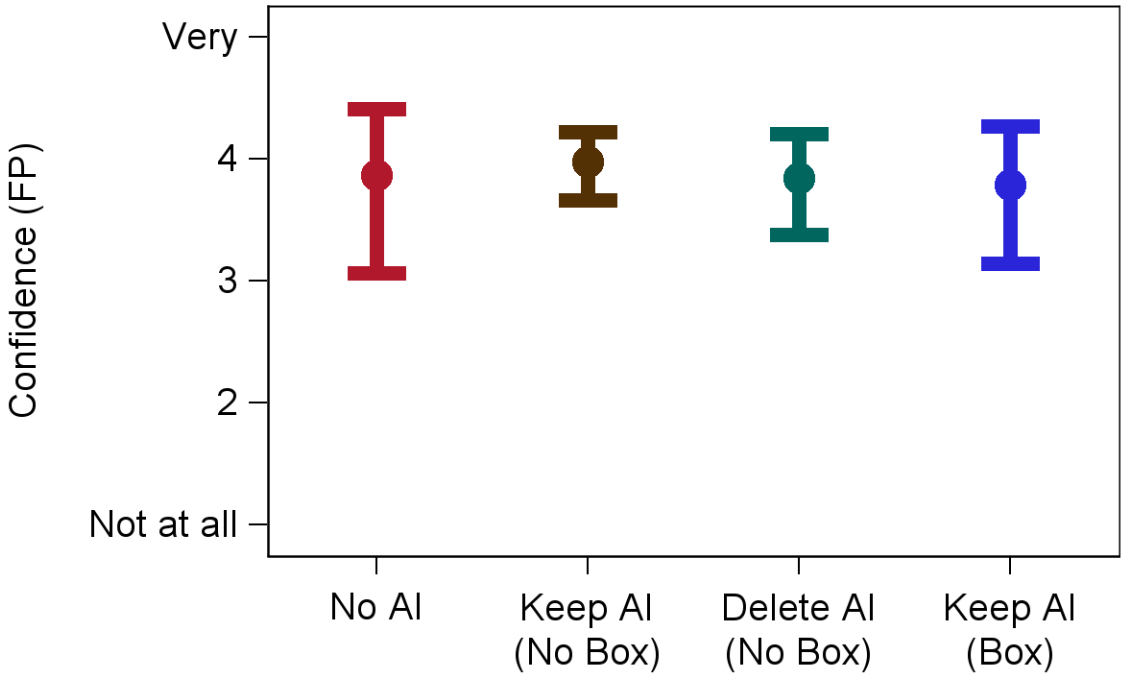

Figure S3a. True Negatives with correct AI feedback by experimental condition
True negative percent (y-axis) is shown for the No AI (Red), Keep AI (No Box) (Brown), Delete AI (No Box) (Green), and Keep AI (Box) (Blue) conditions (x-axis). Mean (circle) and 95% confidence Intervals are displayed.

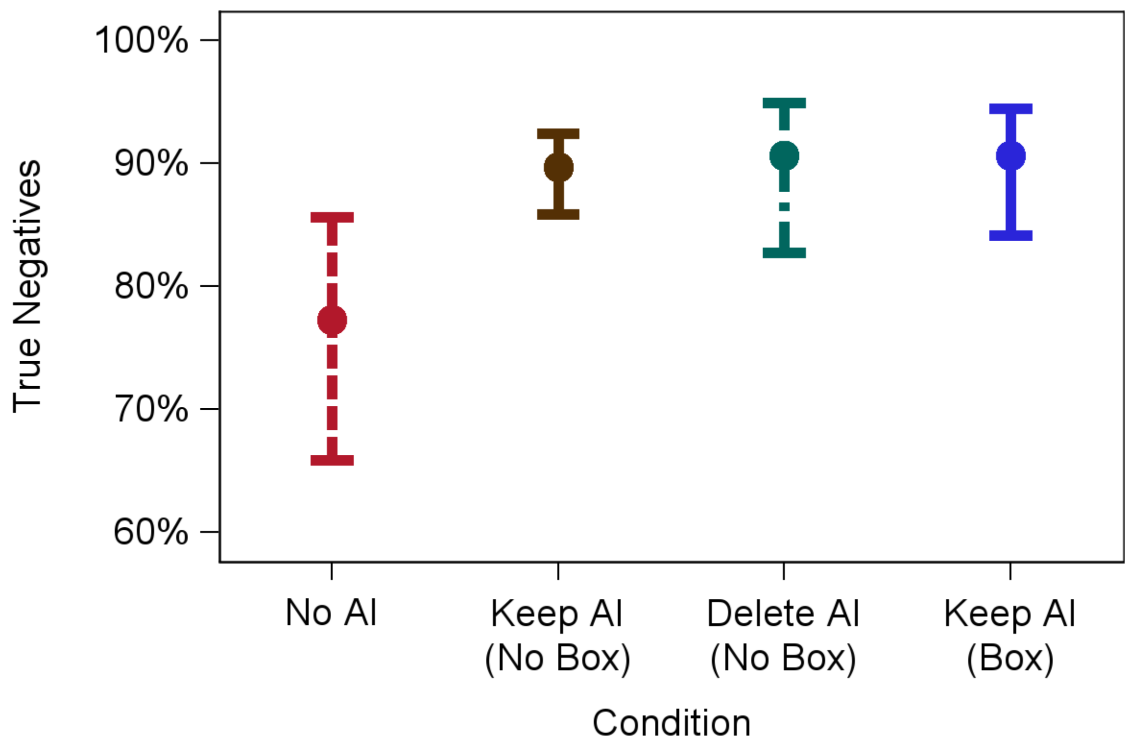

Figure S3b. True Positives with correct AI feedback by experimental condition
True positive percent (y-axis) is shown for the No AI (Red), Keep AI (No Box) (Brown), Delete AI (No Box) (Green), and Keep AI (Box) (Blue) conditions (x-axis). Mean (circle) and 95% confidence Intervals are displayed. Results display the four conditions for the 41 cases where AI provided True Positive results.

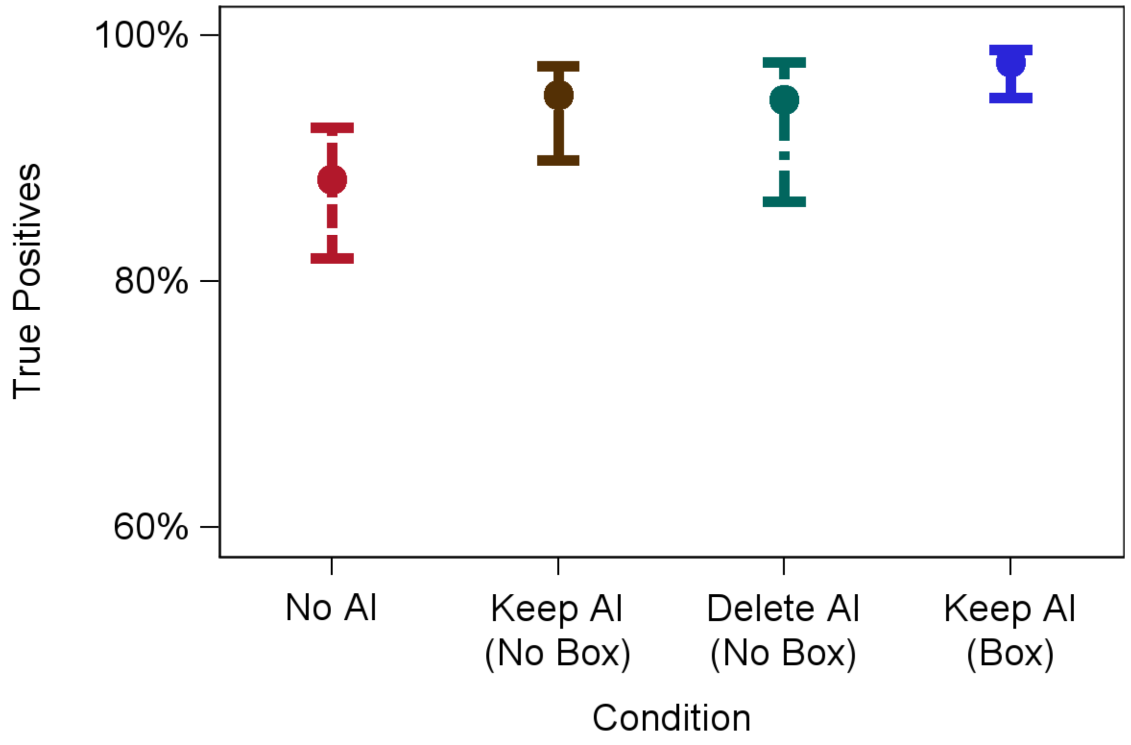

Figure S3c. Confidence: True negative with correct AI feedback.
Confidence rating (from 1=not at all confident to 5=very confident) are shown for the No AI (Red), Keep AI (No Box) (Brown), Delete AI (No Box) (Green), and Keep AI (Box) (Blue) conditions (x-axis). Mean and 95% Confidence Intervals are displayed. TN=True Negative

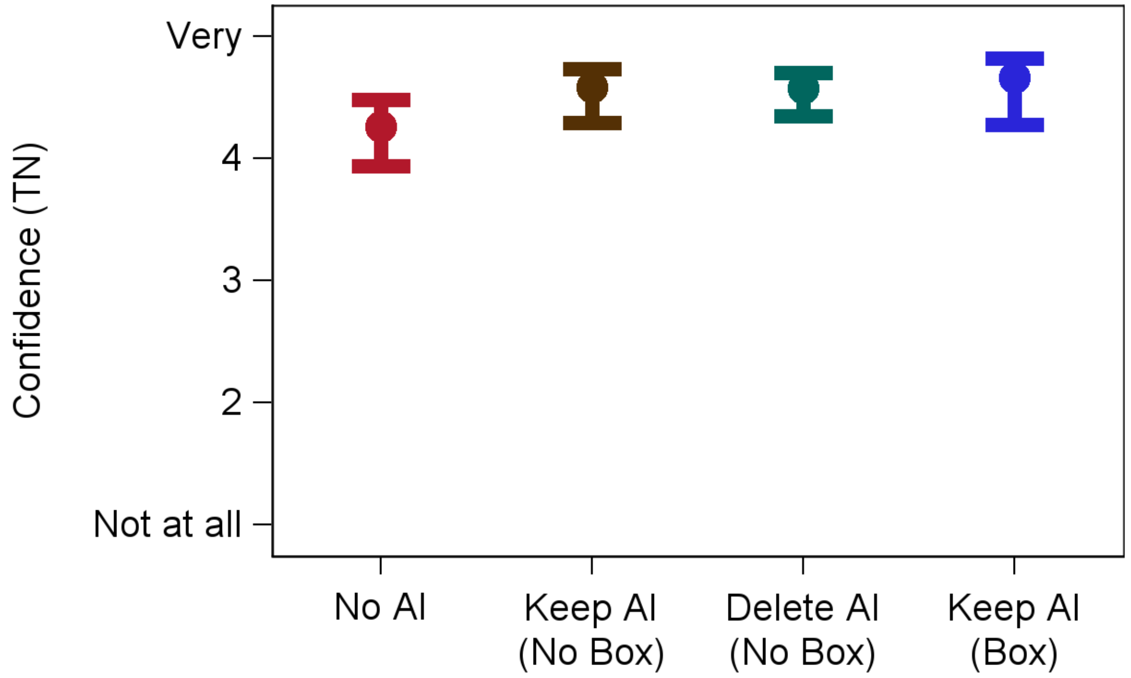

Figure S3d. Confidence: True positive with correct AI feedback.
Confidence rating (from 1=not at all confident to 5=very confident) are shown for the No AI (Red), Keep AI (No Box) (Brown), Delete AI (No Box) (Green), and Keep AI (Box) (Blue) conditions (x-axis). Mean and 95% Confidence Intervals are displayed. TP=True Positive.

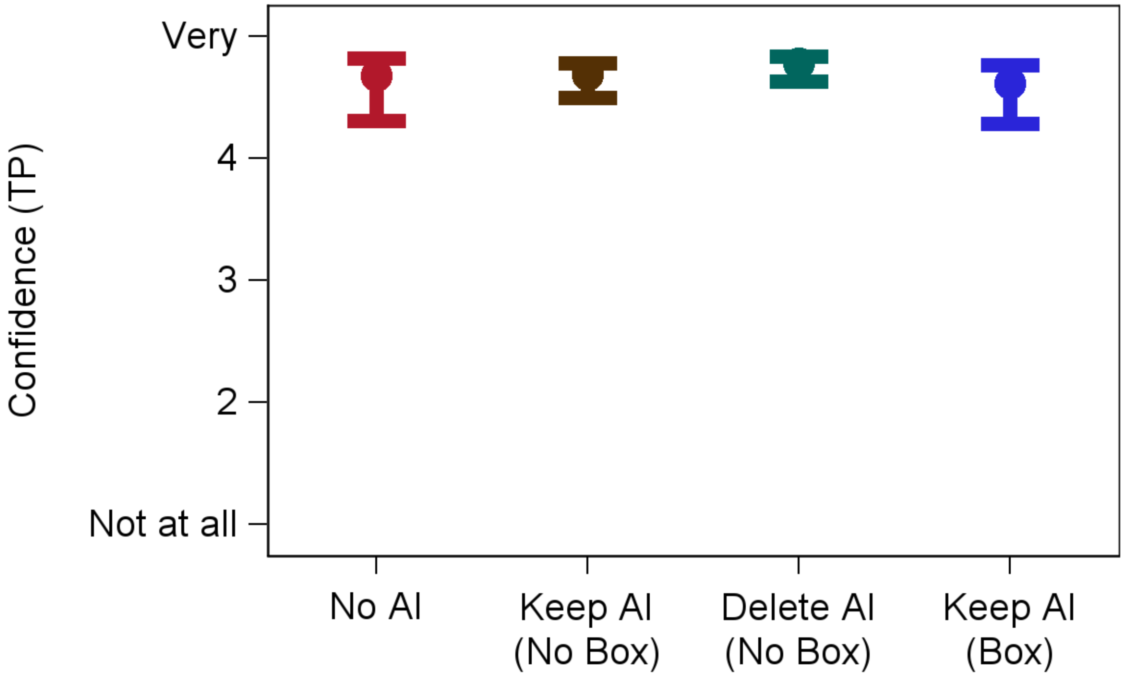

Supplement: Supplementary file 1 — Supplementary file1 (PDF 306 kb) [file 330_2023_9747_MOESM1_ESM.pdf]
